# MicroRNA-10b suppresses the migration and invasion of chondrosarcoma cells by targeting brain-derived neurotrophic factor

**DOI:** 10.3892/mmr.2021.12208

**Published:** 2021-06-08

**Authors:** Abudunaibi Aili, Yong Chen, Hongqi Zhang

Mol Med Rep 13: 441-446, 2016; DOI: 10.3892/mmr.2015.4506

Following the publication of this paper, it was drawn to the Editors’ attention by a concerned reader that certain of the cell Transwell assay data in the article (featured in Figs. 4 and 7) were strikingly similar to data appearing in different form in another article by different authors.

Owing to the fact that the contentious data in the above article had already appeared in different form in another article prior to its submission to Molecular Medicine Reports, the Editor has decided that this paper should be retracted from the Journal. The authors were asked for an explanation to account for these concerns, but the Editorial Office never received any reply. The Editor apologizes to the readership for any inconvenience caused.

